# Cardiac Involvement in Mitochondrial Disorders

**DOI:** 10.1007/s11897-023-00592-3

**Published:** 2023-02-18

**Authors:** Tudor-Alexandru Popoiu, Jan Dudek, Christoph Maack, Edoardo Bertero

**Affiliations:** 1 Department of Translational Research, Comprehensive Heart Failure Center, University Clinic Würzburg, Wurzburg, Germany; 2 “Victor Babes” University of Medicine and Pharmacy, Timisoara, Romania; 3 Present Address: Department of Internal Medicine and Specialties (Di.M.I.), University of Genoa, Genoa, Italy

**Keywords:** Mitochondrial disease, Cardiomyopathy, Mitochondrial DNA, Cardiolipin, Electron transport chain

## Abstract

**Purpose of Review:**

We review pathophysiology and clinical features of mitochondrial disorders manifesting with cardiomyopathy.

**Recent Findings:**

Mechanistic studies have shed light into the underpinnings of mitochondrial disorders, providing novel insights into mitochondrial physiology and identifying new therapeutic targets.

**Summary:**

Mitochondrial disorders are a group of rare genetic diseases that are caused by mutations in mitochondrial DNA (mtDNA) or in nuclear genes that are essential to mitochondrial function. The clinical picture is extremely heterogeneous, the onset can occur at any age, and virtually, any organ or tissue can be involved. Since the heart relies primarily on mitochondrial oxidative metabolism to fuel contraction and relaxation, cardiac involvement is common in mitochondrial disorders and often represents a major determinant of their prognosis.

## Introduction

Mitochondrial disorders are rare genetic diseases, with an estimated prevalence of 10 to 15 cases per 100,000 persons [1], that are characterized by defective mitochondrial oxidative metabolism [2]. Affected genes encode for components of the respiratory chain or proteins involved in mitochondrial DNA replication, mitochondrial dynamics, oxidative metabolism, or the provision of enzyme cofactors. Pathogenic gene variants occur either in mitochondrial or nuclear DNA (mtDNA and nDNA, respectively) and therefore can have any pattern of inheritance — maternal for mtDNA mutations and autosomal or X-linked for nDNA variants. The involvement of certain sets of genes in the development of mitochondrial disorders, including genes encoding fatty acid oxidation enzymes, is currently a matter of debate [3, 4••, 5, 6] [7].

The clinical picture of mitochondrial disorders is extremely heterogeneous, the onset can occur at any age, and any organ or tissue can be involved. Mitochondrial disorders can manifest with a severe multisystemic involvement early in life, often characterized by nonspecific clinical features such as hypotonia, failure to thrive, seizures, and encephalopathy. Alternatively, the disease can present later, from teenage years to adulthood, with a milder, sometimes progressive single-organ or multiorgan involvement. Typical features are exercise intolerance and fatigue, sensorineural hearing loss, and endocrinopathies such as diabetes mellitus. Physiologic stressors such as fever can precipitate cardiac dysfunction and metabolic crisis. The heterogenous clinical picture often makes it difficult to categorize the patient in one definite syndrome. Mutations in the same gene can even cause different disease phenotypes: mutations in the gene encoding the mitochondrial DNA polymerase γ (*POLG*), the polymerase that is responsible for replication and repair of mtDNA, can cause a wide range of phenotypes from early-onset hepatocerebral disease, juvenile epilepsy, or adult ataxia-neuropathy syndrome [8, 9].

Organs that rely primarily on oxidative phosphorylation for adenosine triphosphate (ATP) production, such as the heart and the central nervous system, are frequently involved. Cardiac involvement in mitochondrial disorders is common, and its phenotype and severity are variable [10••]. Both hypertrophic and dilated cardiomyopathy can occur, and the clinical expression ranges from asymptomatic systolic dysfunction to end-stage heart failure requiring heart transplantation in infancy. Left ventricular (LV) non-compaction, an abnormality characterized by the persistence of a prominent trabecular meshwork in the ventricular wall, has been often associated with mitochondrial disorders and can be accompanied by LV dilatation or hypertrophy [11]. Non-compaction probably results from an abnormal morphogenesis of the endomyocardial layer, and its occurrence in mitochondrial disorders might reflect the important role played by mitochondria in cardiac development. Conduction abnormalities have also been associated with mitochondrial disease and are particularly common with certain syndromes, such as Kearns-Sayre.

In this review, we discuss mechanisms and manifestations of cardiac involvement in mitochondrial disorders. We focus on the most common syndromes and on those disorders characterized by a high frequency and severity of cardiac involvement, with a special focus on disorders caused by altered cardiolipin remodelling.

## Pathophysiology of Mitochondrial Disorders

### Mitochondrial DNA

Mitochondria originated from aerobic bacteria that were engulfed by- and evolved in symbiosis with one ancestral anaerobic cell. Mitochondria retain some genes as vestiges of their bacterial progenitors encoded on their mtDNA and machineries for gene transcription and protein translation [12]. The mtDNA is maternally inherited and features a circular DNA formed by two strands, one heavy, guanine-rich strand (H) and one light strand (L). It contains the genetic information for 13 core components of the respiratory chain complexes, 22 transfer RNAs (mt-tRNA), and 2 ribosomal RNAs (mt-rRNA) [13–15]. Therefore, of > 1500 proteins contained in human mitochondria, only 13 are encoded by mtDNA genes, while the remaining are found encoded by the nuclear DNA. Due to the coexistence of 500 or more mtDNA molecules per cell, a phenomenon termed *heteroplasmy,* mitochondrial genetics operate on population-based (instead of Mendelian) principles [16, 17•]. Mitochondrial genes are particular vulnerable to mutations due to the close proximity of mtDNA to sites of reactive oxygen species (ROS) production and lack of protective histones and poor DNA repair mechanisms [13]. Interestingly, the frequency of mitochondrial mutations exceeds by far the incidence of mitochondrial disease, indicating that observable phenotypes only occur when a mutation reaches a certain threshold [18]*.* While the mechanisms governing mitotic segregation of mtDNA are poorly understood, this process might not result uniquely from random genetic drift — on the contrary, experimental evidence demonstrates the occurrence of tissue-specific and age-related directional selection for certain mtDNA genotypes in the same organism [19]. Moreover, in circulating blood cells, a selective recognition of mtDNA by the innate immune system has been observed [20]. As a result of these factors, the wild type-to-mutant mtDNA ratio varies from tissue to tissue and even from cell to cell [21, 22•]. The threshold at which one mtDNA mutation results in cellular defects depends on the mutation itself and the cell type. The high reliance of the heart and central nervous system on aerobic metabolism might decrease the proportion of mutant mtDNA required for disease manifestation to arise [7].

### ATP and Reactive Oxygen Species Production at the Respiratory Chain

**Fig. 1:**
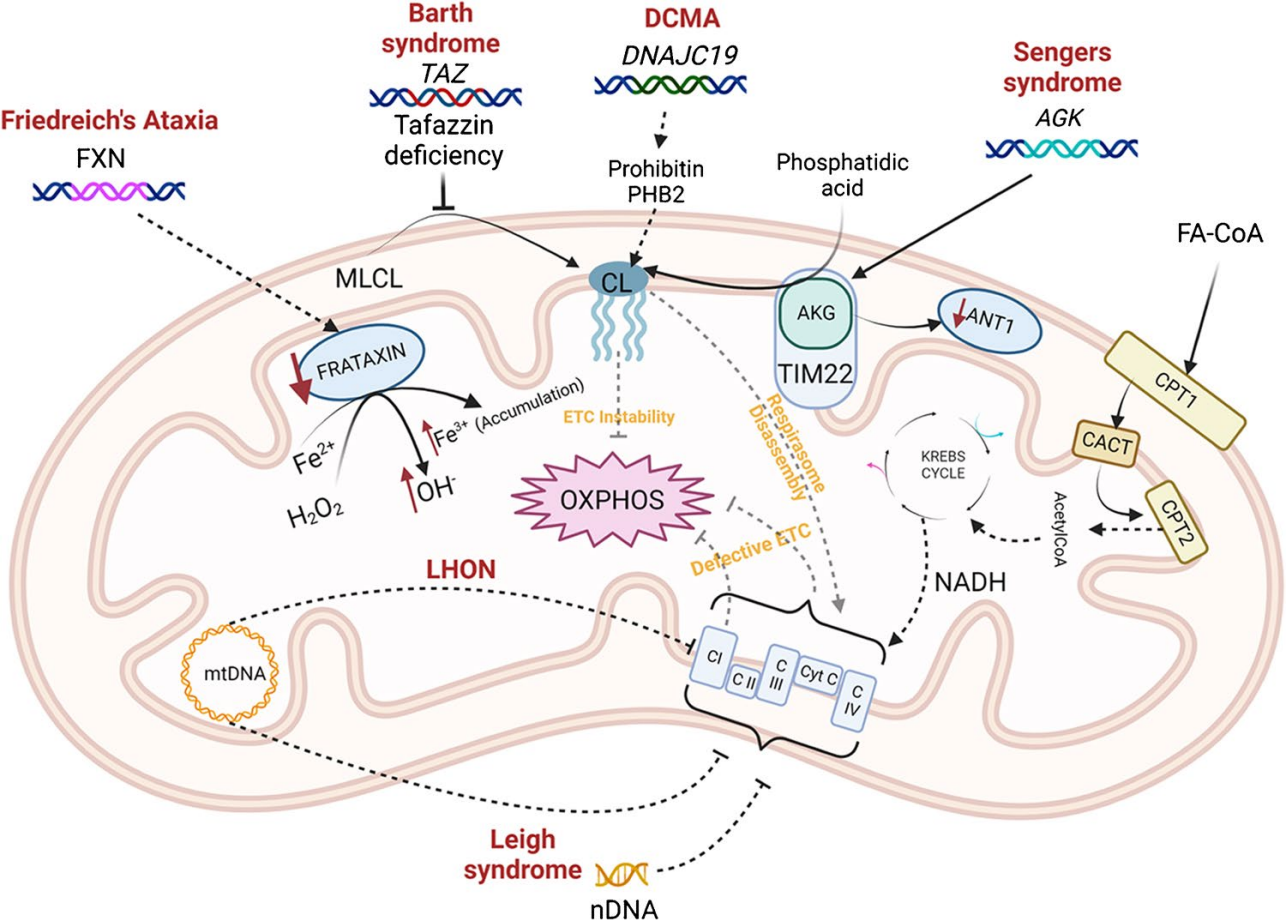
Mechanisms of mitochondrial dysfunction in inherited mitochondrial disorders. Created with BioRender.com. Abbreviations: AGK, acylglycerol kinase; ANT1, adenine nucleotide translocator 1; CACT, carnitine-acylcarnitine translocase; CL, cardiolipin; CPT1/2, carnitine palmitoyltransferase 1/2; DCMA, dilated cardiomyopahty with ataxia; ETC, electron transport chain; FA-CoA, fatty acyl-CoA; *FXN*, frataxin; LHON, Leber’s hereditary optic neuropathy; OXPHOS, oxidative phosphorylation; *TAZ*, tafazzin

One central function of mitochondria is the production of adenosine triphosphate (ATP) via oxidative phosphorylation. This process entails the transfer of electrons across a series of protein complexes embedded in the inner mitochondrial membrane, the electron transport chain complexes (ETC), which harness the energy derived from sequential oxidation steps to pump protons from the mitochondrial matrix to the intermembrane space (Fig. 1). The resulting chemical and electrical potential, termed the protonmotive force, is used by the F_1_-F_o_ ATP synthase to catalyse ADP phosphorylation and drives many essential mitochondrial functions such as metabolite, ion, and protein transport across the inner mitochondrial membrane [24, 25]. Organs with a very high ATP demand, such as the heart and the central nervous system, mainly rely on oxidative phosphorylation to maintain their function, and the failure to generate sufficient ATP is considered the primary driver of disorders caused by mutations in the mtDNA or in nuclear genes encoding respiratory chain components [26].

Molecular oxygen (O_2_) is the final acceptor of electrons at the respiratory chain. The high affinity of O_2_ for electrons explains the large amount of energy released by the process of electron transfer at the ETC. Incomplete reduction of O_2_, however, can lead to the production of the highly reactive superoxide radical (O_2_^−^), which has a strong tendency to subtract electrons from proteins, lipids, and nucleic acids. To protect the cell from the potentially devastating consequences of mitochondrial oxidative stress, mitochondria are equipped with an efficient antioxidant machinery [27]. First, superoxide is converted to hydrogen peroxide (H_2_O_2_) by the manganese-dependent superoxide dismutase (Mn-SOD). Subsequently, H_2_O_2_ is reduced to H_2_O by matrix peroxidases, including peroxiredoxin (Prx) and glutathione peroxidases (Gpx). The reducing equivalents required for H_2_O_2_ elimination by Prx and Gpx derive from a cascade of redox reactions that are replenished by the activity of the Krebs cycle [28]. Therefore, mitochondrial oxidative metabolism is essential both for ATP production at the ETC and protect the cell from mitochondrial ROS-induced damage. As mitochondrial ROS play a role as a signalling molecule, changes in ROS can also affect signalling pathways to control metabolism, proliferation, and differentiation [29]. Therefore, a tight control of ROS is important to maintain cellular and organismal homeostasis. Oxidative stress has been implicated in the pathophysiology of many mitochondrial disorders, a notion that has been mostly supported by in vitro models of disease [30]. In principle, any mutation affecting the structure and function of the ETC can increase mitochondrial superoxide production at the respiratory chain, overwhelming mitochondrial antioxidant systems.

Besides their role in energy and redox homeostasis, mitochondria also play a central role in synthesizing amino acids, enzyme cofactors such as heme, and iron-sulphur clusters, and are central players in cellular ion handling [31]. Mitochondria are essential modulators of Ca^2+^ dynamics as membrane-bound Ca^2+^ pumps are ATP- and ROS-dependent [32]. In turn, Ca^2+^ concentrations in the mitochondrial matrix modulate oxidative metabolism by regulating the activity of the rate-limiting enzymes of the Krebs cycle [28, 33].

## Disorders Caused by mt-tRNA Mutations

### MELAS and MERRF

Mitochondrial encephalopathy, lactic acidosis, and stroke-like episodes (MELAS) and myoclonic epilepsy with ragged red fibres (MERRF) are predominantly caused by pathogenic mutations in genes encoding for mt-tRNA, albeit rare cases associated with mutations in mt-rRNA genes were described. The most common (~ 80%) pathogenic mutation found in patients with MELAS involves the transition from adenine to guanine at position 3243 (m.3243A > G), which disrupts the mitochondrial leucyl-tRNA gene, *MT-TL1*. The consequent defect in mitochondrial oxidative metabolism uncouples glycolysis from oxidation of pyruvate, which is therefore converted into lactate leading to lactic acidosis [34–36]. Mutations in the mitochondrial lysil-tRNA gene *MT‐TK* account for more than 90% of MERFF cases [37]. Both MELAS and MERFF are polygenic disorders, and mutations causing MELAS can also give rise to different phenotypes, such as Leigh syndrome and chronic progressive external ophthalmoplegia [22•].

Three key features are deemed necessary for the diagnosis of MELAS: (i) stroke-like episodes before the age of 40; (ii) encephalopathy characterized by seizures, dementia, or both; and (iii) lactic acidosis, presence of ragged-red fibres, or both [38]. The spectrum of central and peripheral nervous system involvement is wide and includes cognitive impairment in infancy, visual or auditory sensual deficiencies, and peripheral neuropathy. Furthermore, endocrine manifestations such as diabetes mellitus, renal involvement in the form of Fanconi disease, and gastrointestinal abnormalities have been associated with MELAS [39].

Cardiac involvement in MELAS occurs in ~ 55% of patients and manifests with cardiomyopathy and/or conduction defects [40•]. Hypertrophic cardiomyopathy is more common than dilated cardiomyopathy, and both phenotypes can present in association with LV non-compaction. Conduction defects include ventricular pre-excitation and atrio-ventricular block. A meta-analysis assessing the frequency of cardiac involvement in mitochondrial myopathies found that patients with MELAS have the highest prevalence of EKG and echocardiographic abnormalities compared with other mitochondrial disorders, and cardiac manifestations in infancy portend a more severe phenotype with an increased mortality rate [40•, 41••].

MERFF is a neuromuscular disorder with multisystemic involvement varying from myoclonus, cerebellar ataxia, dementia, and muscle weakness to peripheral neuropathy, respiratory dysfunction, cardiomyopathy, and lipomas [42]. Cardiac involvement occurs in 44–57% of MERFF cases [43, 44], and hypertrophic and dilated cardiomyopathy appear to be equally common [45]. Prevalence of arrhythmic events seems to be lower than in MELAS, the most common being Wolff-Parkinson-White syndrome [44].

## Disorders Caused by Defects in the Electron Transport Chain

### Leigh Syndrome

Leigh syndrome or subacute necrotizing encephalopathy is the most common presentation of a mitochondrial disease in infancy and typically caused by mutations in at least 75 different mtDNA and nDNA genes. Mutations in mtDNA account for 20–25% of Leigh syndrome cases and affect the function of either complex I or complex V of the respiratory chain, while the remaining 75–80% result from nDNA mutations, the majority of which lead to decreased protein levels and/or activity of cytochrome *c* oxidase (complex IV) [46].

Mice lacking the complex I subunit *Ndufs4* (*Ndufs4*^−/−^ mice) exhibit an extensive remodelling of metabolic pathways driven by the activation of the metabolic sensor mechanistic target of rapamycin (mTOR). Inhibition of mTOR with rapamycin delayed disease progression in *Ndufs4*^−/−^ mice [47, 48]. In addition, loss of complex I activity leads to accumulation of NADH, resulting in an increased NADH/NAD^+^ ratio. Because deacetylation is a NAD^+^-dependent process, the shift in the NADH/NAD^+^ ratio results in hyperacetylation and consequent functional changes in numerous proteins, including the cardiac sodium channel Na_V_1.5. On these grounds, supplementation with the NAD precursor nicotinamide riboside (NR) was proposed as a therapeutic strategy to prevent arrhythmia in Leigh syndrome [49].

Leigh syndrome is characterized by progressive cognitive decline, hypotonia, psychomotor disability, ataxia, and high levels of lactate detectable both in blood and cerebrospinal fluid [50, 51]. Disease onset is usually at 2 years of age, reaching peak mortality towards the age of 3. Neurogenic muscle weakness, ataxia, and retinitis pigmentosa (NARP), a disorder characterized by a later onset and dominated by ataxia and peripheral neuropathy, is considered a clinical continuum with maternally inherited Leigh syndrome. The latter, however, is far more severe and lethal, a difference that might be explained by the higher proportion of mutant mtDNA observed in NARP [51, 52].

Cardiac involvement represents the most common non-neurological manifestation of the disease [53]. In a multi-centric study involving 96 patients with Leigh syndrome, cardiac involvement in the form of cardiomyopathy, arrhythmia, conduction defects, or valve disease was identified in 18 patients. Cardiomyopathy, both hypertrophic and dilated, was diagnosed in 10% of the cohort and more frequent among patients carrying mtDNA mutations than in those with nDNA mutations [54•].

### Leber’s Hereditary Optic Neuropathy

Leber’s hereditary optic neuropathy (LHON) is a maternally inherited disorder that in 95% of cases is caused by one of three mutations in mtDNA genes encoding subunits of complex I of the respiratory chain [55]. Complex I dysfunction leads to increased ROS emission and defective ATP production, which are considered the main drivers of disease progression. Oxidative stress and bioenergetic deficit cause cell death by triggering the opening of a large pore in the mitochondrial membranes, the permeability transition pore, which irreversibly dissipates the proton-motive force and releases proapoptotic factors into the cytosol [55, 56, 57].

For yet unknown reasons, the penetrance of LHON is higher in men than in women (45% vs 10%, respectively). Environmental factors, such as smoking and alcohol consumption, appear to double the risk of disease manifestation [58•, 59]. The typical presentation is subacute, one-sided loss of vision occurring in the absence of pain, starting with a centrally located scotoma that progressively involves the entire visual field [55, 56, 60]. Although single-organ involvement is common, cardiac and myopathic manifestations have also been reported. Among cardiac manifestations, arrhythmias and conduction defects have the highest prevalence (15–50% of the cases) [60, 61].

### Kearns-Sayre Syndrome

Kearns-Sayre syndrome (KSS) is caused by large mtDNA deletions that give rise to progressive external ophthalmoplegia and retinitis pigmentosa before 20 years of age. In addition to these two features, at least one between cardiac conduction defects, cerebellar ataxia, or increased protein levels in the cerebrospinal fluid (> 100 mg/dL) is required to establish diagnosis [62]. KSS involves the heart in ~ 50% of cases and primarily affects the cardiac conduction system [63]. Conduction abnormalities usually occur after the onset of ocular involvement and can present with syncope or sudden cardiac death. Severe forms of dilated cardiomyopathy have also been described in KSS [64–66].

## Disorders Caused by Defective Cardiolipin Remodelling

### Cardiolipin Function

The inner mitochondrial membrane (IMM) has the highest density of embedded proteins among cellular membranes. The integrity, fluidity, and phospholipid composition of the IMM is pivotal to maintain the architecture and function of membrane-embedded protein complexes, including the ETC. Cardiolipin (CL) is a phospholipid that is almost exclusively found in the IMM, where it plays a role in numerous key mitochondrial processes by interacting with several IMM protein complexes. For instance, CL stabilizes the ETC in larger macromolecular complexes termed “respirasomes,” facilitating the electron transfer process and thereby, maintaining respiratory efficiency or “coupling.” Furthermore, CL is abundant near the tips of the mitochondrial cristae, where it contributes to the curvature of the IMM and favours ATP synthase dimerization to maximize ATP synthesis [67]. CL is formed by a polar glycerol head to which four fatty acyl chains are attached. After biosynthesis, CL undergoes a process known as remodelling, which involves the substitution of the saturated acyl chains of “premature” CL with polyunsaturated acyl species. The different CL composition might account for a more or less condensed architecture of mitochondrial cristae in different tissues [70, 71•]. Furthermore, the process of CL remodelling is pivotal to allow the interaction of CL with proteins and the crowding of proteins in the IMM.

Mitochondrial disorders caused by abnormal CL composition are caused by nDNA mutations in genes encoding enzymes involved in CL biosynthesis and remodelling. This group of disorders includes at least three syndromes, i.e., Sengers syndrome, dilative cardiomyopathy with ataxia (DCMA), and Barth syndrome, whose dominant features are cardiomyopathy and skeletal myopathy. These disorders were initially classified together with inborn errors of metabolism characterized by increased urinary 3-methylglutaconic acid excretion, which is also observed in a minority of patients with other disorders of oxidative phosphorylation, but is especially common in Barth syndrome and DCMA. Overall, cardiomyopathy is a hallmark of disorders caused by defective CL remodelling, demonstrating the pivotal role of this phospholipid for mitochondrial function in the heart [68•, 69].

### Barth Syndrome

Barth syndrome is caused by mutations in the gene encoding tafazzin [72], a mitochondrial transacylase ubiquitously expressed in human tissues which catalyses the final step in the remodelling of CL [73]. In Barth syndrome, abnormal CL remodelling alters CL-protein interaction, causing the accumulation of more saturated CL species and accelerating CL turnover [74].

Barth syndrome is characterized by the infantile onset of cardiomyopathy, skeletal myopathy, recurrent neutropenia, and retarded growth [75]. The most common cause of death in Barth syndrome is cardiomyopathy, which can manifest as hypertrophic or dilated cardiomyopathy, often (50%) associated with LV noncompaction. Cardiac involvement can present with severe LV dysfunction and heart failure in the first years of life [76]. A sizable proportion of Barth syndrome patients, however, develop a milder form of cardiomyopathy, with preserved (i.e., > 50%) or only mildly reduced (40–50%) LV ejection fraction (LVEF) and impaired LV relaxation. Overall, patients developing a reduced LVEF are more prone to cardiovascular events, including arrhythmias and sudden cardiac death, whereas those with preserved LVEF are mainly symptomatic because of exercise intolerance and fatigue [76, 77]. These symptoms have been attributed to the marked reduction in cardiac contractile reserve and impaired skeletal muscle oxidative metabolism in Barth syndrome [78].

Mechanistic insights into the pathophysiology of Barth syndrome cardiomyopathy were obtained by two different mouse models of the disease, the tafazzin-knockdown (*Taz*-KD) and -knockout (*Taz*-KO) models. While *Taz*-KO mice are characterized by embryonic lethality, impaired growth, and dilated cardiomyopathy with severe systolic dysfunction [79], *Taz*-KD exhibit only a mild reduction in LVEF and a limited inotropic reserve, recapitulating the phenotype of those Barth syndrome patients who do not develop (or recover from) severe HF during infancy [80••].

**Fig. 2:**
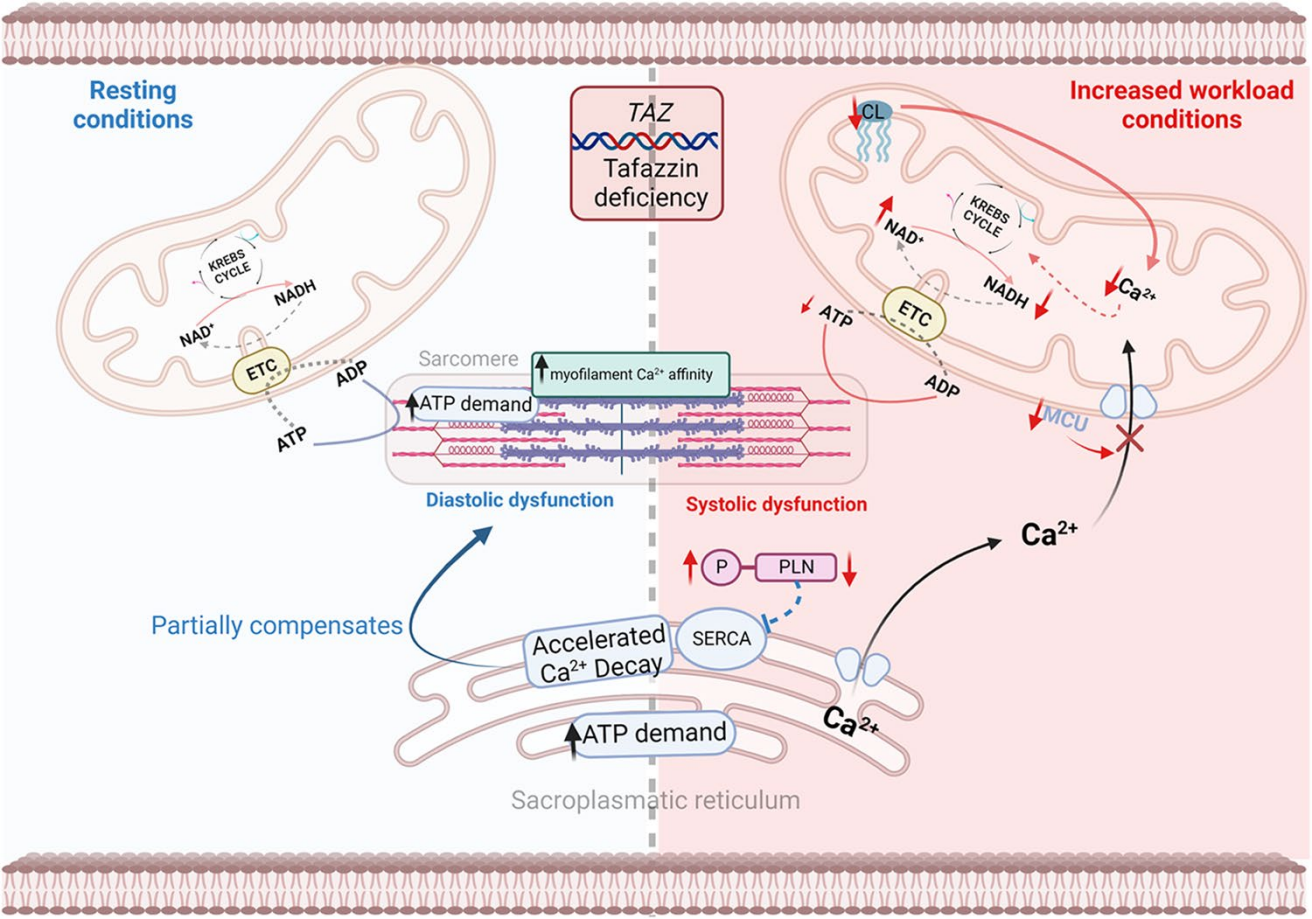
Defects in cardiac excitation–contraction coupling and cellular calcium handling in Barth syndrome. Created with BioRender.com. Abbreviations: ETC, electron transport chain; MCU, mitochondrial calcium uniporter; *TAZ,* tafazzin

In the *Taz*-KD model, we discovered that altered CL remodelling causes not only changes in the respiratory chain but also alters the structural organization of the mitochondrial Ca^2+^ uniporter, a Ca^2+^ channel embedded in the IMM [80••]. These structural changes hinder mitochondrial calcium (Ca^2+^) uptake. Under physiological conditions, Ca^2+^ accumulation in the mitochondrial matrix stimulates the Krebs cycle dehydrogenases, thereby accelerating ATP production when an elevation in cardiac workload increases ATP consumption in the cytosol [81]. In Barth syndrome, the Ca^2+^-dependent stimulation of mitochondrial oxidative metabolism is lost, contributing to the reduced contractile reserve and the consequent exercise intolerance (Fig. 2). In addition, *Taz*-KD mice also exhibit elevated myofilament Ca^2+^ affinity and slowed cross-bridge cycling, which hinders myocardial relaxation and imposes an additional ATP demand during diastole. Although evidence of diastolic dysfunction in patients is scant, these results indicate that impaired myocardial relaxation might contribute to the manifestation of cardiomyopathy in Barth syndrome [80••].

Investigations in rodent models of the disease have also revealed two distinct and possibly complementary mechanisms explaining the increased susceptibility to arrhythmias and sudden cardiac death in Barth syndrome [82]. First, the defect in mitochondrial Ca^2+^ uptake may contribute to this risk by causing mitochondrial oxidation that triggers arrhythmias in isolated cardiac myocytes and slows conduction in whole hearts [80••]. Furthermore, in the *Taz*-KO model, high levels of mitochondrial ROS activate the Ca^2+^/calmodulin-dependent protein kinase II (CaMKII), which phosphorylates the ryanodine receptor 2 (RyR2), i.e., the Ca^2+^ channel that mediates Ca^2+^ efflux from the sarcoplasmic reticulum. RyR2 phosphorylation leads to spontaneous Ca^2+^ release events from the sarcoplasmic reticulum, altering cellular Ca^2+^ handling and causing afterdepolarization events that represent a trigger for ventricular arrhythmias [83].

One unresolved issue concerns the role of oxidative stress in the pathogenesis of Barth syndrome cardiomyopathy*.* In vitro studies implicated ROS production at the ETC as one key driver of the disease [84, 85], which is in line with observations in the *Taz*-KO model. In contrast, *Taz*-KD mice do not display signs of oxidative stress in the heart, and genetic or pharmacological interventions targeting ROS production did not rescue cardiac dysfunction in this model [80••, 86]. In Barth syndrome patients, treatment with the small peptide elamipretide (also known as SS-31, MTP-131 or Bendavia), which prevents ROS formation at the ETC by selectively targeting CL, did not meet the primary endpoint in a 4-week, double-blind, placebo-controlled crossover trial, but it did improve physical performance in the trial open-label extension [87].

Overall, the *Taz*-KD and *Taz*-KO models exhibit remarkable differences in their cardiac phenotype that might reflect the heterogeneity of disease manifestations and mechanisms at play in Barth syndrome cardiomyopathy. Future investigations should be aimed at addressing the underpinnings of this heterogeneity, which probably involves interactions of tafazzin mutations with the genetic background of the patients.

### Sengers Syndrome

Sengers syndrome is an autosomal recessive disorder caused by mutations in the gene encoding acylglycerol kinase (AGK) [88]. AGK is located within the IMM and has two known functions: it is a phospholipid kinase that synthesizes phosphatidic acid, a precursor of CL, and a component of the membrane translocase complex TIM22, which mediates the import of mitochondrial membrane proteins that are synthesized in the cytosol. This moonlight function of AGK might explain the wide spectrum of mitochondrial abnormalities observed in Sengers syndrome [89, 90]. One prominent defect is decreased activity of the mitochondrial adenine nucleotide translocator (ANT1), which was initially considered the primary cause of the disease.

The clinical picture of Sengers syndrome is characterized by congenital cataracts, hypertrophic cardiomyopathy, skeletal myopathy, and lactic acidosis [88]. Similar to Barth syndrome, Sengers syndrome can present with an extremely severe form of hypertrophic cardiomyopathy in newborns, which portends an extremely poor prognosis, or as a late-onset, milder form with a life expectancy exceeding the fifth decade of life [91–93].

### Dilated Cardiomyopathy with Ataxia

DCMA is an autosomal recessive monogenic disorder caused by variants in the *DNAJC19* gene, which were identified and are especially frequent in the Canadian Dariusleut Hutterite population [94, 95]. Most pathogenic variants in *DNAJC19* are truncating, and thus result in a shorter version of the protein [94, 96, 97]. DNAJC19 is a component of the presequence protein translocase and shows sequence similarity with the yeast J domain protein Pam18/Tim14 [98•, 99]. Besides its role in mitochondrial protein transport, DNAJC19 was also found to interact with membrane proteins of the prohibitin family [100]. Prohibitins form a large complex in the inner membrane, which serves as a structural scaffold involved in CL metabolism. DNAJC19-deficient cells show altered CL levels [100]. Therefore, akin to Barth syndrome, the clinical picture of DCMA arises from abnormalities in the CL composition in the IMM [100, 101].

DCMA manifests with early-onset dilated cardiomyopathy, conduction defects, growth failure, ataxia, male genital anomalies, and developmental defects. LV non-compaction and oculomotor apraxia have also been associated with DCMA [102]. Cardiac involvement is often severe, with a reported 50% mortality due to congestive heart failure or arrhythmias at the mean age of 22 months [103].

## Disorders Caused by Defective Fatty Acid Oxidation

Fatty acid oxidation disorders comprise defects in fatty acid import in the mitochondrial matrix via the carnitine shuttle and abnormalities in mitochondrial β-oxidation (Fig. 1). Disease manifestations are attributed to defective mitochondrial ATP production, and therefore, certain features recapitulate those of other mitochondrial disorders [104–108]. Moreover, defects in fatty acid oxidation can induce extensive metabolic remodelling causing increased systemic demand for pyruvate and trigger stress responses due to altered amino acid metabolism [109]. However, one additional pathogenic mechanism that is specific for fatty acid oxidation disorders is the accumulation of toxic long-chain CoA-esters or their free long-chain fatty acids, which perturb cellular function by lowering cytosolic pH, suppressing intermediary metabolism, or causing oxidative stress via lipid peroxidation [110••, 111].

Cardiomyopathy and metabolic crisis secondary to liver failure are among the most severe and precocious manifestations of these disorders, whereas exercise intolerance and rhabdomyolysis may appear later in infancy [106]. Disease onset might be abrupt and can be elicited by a variety of stressors. Late-onset CPT2 deficiency is the most common genetic cause of rhabdomyolysis in adults, and exercise is the most frequent trigger of rhabdomyolysis in these patients. Other triggers of metabolic decompensation include viral infections, fasting, colds, general anaesthesia, and sleep deprivation [112].

### Friedreich’s Ataxia

Friedreich’s ataxia (FRDA) is the most prevalent cause of hereditary ataxia among individuals of European descent, affecting around 1 in 40,000 subjects. FRDA is an autosomal recessive disorder that usually manifests between 10 and 15 years of age. Its symptoms include progressive ataxia, dysarthria, vision and hearing loss, muscle weakness, musculo-skeletal abnormalities such as scoliosis [113], diabetes mellitus, and hypertrophic cardiomyopathy [114]. The cause is a homozygous GAA triplet repeat in the first intron of the *FXN* gene, which encodes for the mitochondrial protein frataxin [115]. *FXN* alleles contain > 70 GAA repeats that interfere with gene transcription by heterochromatin silencing, leading to loss of frataxin protein.

Frataxin is a mitochondrial iron-binding protein that is crucially involved in the assembly and repair of iron-sulphur clusters which operate as cofactors in a number of fundamental cellular processes, including oxidative phosphorylation, the Krebs cycle, and enzymes such as aconitase [116]. Accordingly, endomyocardial biopsies of FRDA patients demonstrated a reduced activity of mitochondrial respiratory chain complexes I, II and III. It has been proposed that the ferroxidase activity of frataxin might help reduce the concentration of chelatable iron and prevent this metal from producing ROS [117]. Deregulation of the antioxidant transcription factor nuclear factor-E2-related factor-2 (NRF2) and reduced expression of peroxiredoxins, glutaredoxins, and glutathione S-transferase causes depletion of antioxidants and, consequently, an increase in lipid peroxidation[118, 119]. Lipid peroxidation resulting from iron accumulation and depletion of antioxidant systems are crucial triggers of ferroptosis, a type of programmed cell death [120]. In fact, fibroblasts derived from FRDA patients and from FRDA mouse models are hypersensitive to drug-induced ferroptosis and rescued by specific ferroptosis inhibitors [121].

Although central nervous system involvement dominates the clinical presentation of FRDA, cardiovascular involvement dictates its prognosis, accounting for ~ 59% of deaths among FRDA patients [122]. The prognosis is particularly poor for those with progressive LV systolic dysfunction [123]. Cardiac involvement in FRDA is characterized by concentric LV hypertrophy occurring without outflow tract obstruction and with an end-diastolic wall thickness < 15 mm in the majority of cases [124, 125•].

One longitudinal study with a 10-year follow-up period described two distinct cardiac trajectories in FRDA patients: a larger low-risk group with normal LV systolic function at baseline, which slightly decreased over time but remained within normal range, and a smaller high-risk group exhibiting a progressive decline in LVEF. Of note, there was a positive correlation between the size of the expanded GAA repeat and the risk of cardiac complications [126]. Over time, wall thickness tends to decrease and LV dilatation ensues, and ultimately ~ 20% exhibit a reduced LVEF [127]. In advanced stages of the disease, the onset of atrial fibrillation can additionally deteriorate systolic function and lead to heart failure [122].

Late-onset (LOFA, onset > 25 years) and very late-onset FRDA (VLOFA, onset > 40 years) were described as rare variants of the disease. FRDA cases with delayed onset exhibit a milder phenotype, a slower rate of disease progression, and a more diverse spectrum of signs and symptoms. Akin to “typical” FRDA, the most frequent presenting signs are gait and limb ataxia, but dysarthria often appears later and spasticity is more common, while the prevalence of non-neurologic symptoms such as cardiomyopathy or diabetes mellitus is lower [128]. At the other end of the spectrum, patients with early onset of FRDA (< 5 years of age) exhibit a more severe phenotype and a poorer prognosis. Again, the size of the expanded GAA repeat correlates with the onset and severity of the clinical presentation [129].

## Conclusions

Cardiac involvement in mitochondrial disorders is common and often severe. In spite of the advances in the understanding of the mechanism of disease, many of these disorders are orphan of treatment and their prognosis remains poor. Only few therapeutic agents are approved for the treatment of mitochondrial diseases [130]. This unmet medical need has stimulated research for novel disease mechanism and novel therapeutic options. Development of zinc-finger nucleases (ZFNs), transcription activator-like effector nuclease (TALENS), and Crispr-Cas9 allows manipulation of the mitochondrial genome [131, 132]. More research is necessary for the development of innovative treatments for rare mitochondrial disorders.
